# Machine-learning-based ground sink susceptibility evaluation using underground pipeline data in Korean urban area

**DOI:** 10.1038/s41598-022-25237-8

**Published:** 2022-12-03

**Authors:** Jun Hwan Park, Junggoo Kang, Jaemo Kang, Duhwan Mun

**Affiliations:** 1 School of Mechanical Engineering, Korea University, 145 Anam-ro, Seongbuk-gu, Seoul, 02841 Republic of Korea; 2 Department of Geotechnical Engineering Research, Korea Institute of Civil Engineering and Building Technology, 283 Goyang-daero, Ilsanseo-gu, Goyang-si, 10223 Goyeonggi-do Republic of Korea

**Keywords:** Civil engineering, Computer science

## Abstract

Ground subsidence caused by natural factors, including groundwater, has been extensively researched. However, there have been few studies on ground sink caused mainly by artifacts, including underground pipelines in urban areas. This paper proposes a method of predicting ground sink susceptibility caused by underground pipelines. Underground pipeline data, drilling data, and 77 points of ground sink occurrence were collected for five 1 × 1 km urban areas. Furthermore, three ground sink conditioning factors (GSCFs) (pipe deterioration, diameter, and length) were identified by correlation analysis. Pipe deterioration showed the highest correlation with ground sink occurrence, followed by pipe length and pipe diameter in that order. Next, four machine learning methods [multinomial logistic regression (MLR), decision tree (DT) classifier, random forest (RF) classifier, and gradient boosting (GB) classifier] were applied. The results show that GB classifier had the highest accuracy of 0.7432, whereas the accuracy of RF classifier was 0.7407; thus, GB classifier was not significantly more accurate. RF classifier showed the highest reliability (0.84, 0.70, 0.87) according to the area under the receiver operating characteristic (AUC–ROC) curve. Ground sink susceptibility maps (GSSMs) of the five regions in an urban area were created using RF classifier, which performed the best overall.

## Introduction

Ground sink is a geological disaster with various causes. It has caused large amounts of damage in urban areas, for example, Seoul^1^, Jeong-am^2^, Beijing^3^, Lazio^4^, Al Ain^5^, and Perak^6^. In South Korea, 169 ground sinks occurred in the past seven years in Seoul^7^, and 3328 ground sinks reportedly occurred between 2010 and 2014^8^. However, most of the accidents that occurred in South Korea were caused by artifacts rather than sinkholes resulting from natural factors, and the term “sinkhole” is often misused. Ground sink is an academic term for a phenomenon in which the surface collapses very rapidly for several reasons and exhibits local movement downward, as defined by the Ministry of Land, Infrastructure and Transport^9^. Phenomena similar to ground sink include ground subsidence, in which the ground slowly sinks for a long time because of natural or artificial factors, and sinkholes, where layers such as limestone, gypsum, and rock salt are lost and collapse owing to chemical effects^9^. Sinkholes, which are often confused with ground sinks, are related mainly to the composition of limestone in the ground as well as groundwater movement. Cavities are generated as the limestone layer is eroded by groundwater, which eventually causes the cavities to collapse^4,10^. Factors that can cause ground sink include groundwater exploitation, irrigation, mining, and subway development^2,3,5,11^. Ground sink that occurs outside of urban areas generally does not cause extensive damage. However, when ground sink occurs in an urban area, infrastructure such as underground utilities, roads, bridges, and railways can be damaged, possibly causing environmental, social, and economic harm^12^.

Many studies have used machine learning algorithms to predict ground subsidence. For example, Taheri et al.^11^ used four Bayes-based machine learning algorithms. They reported that water level decline and the penetration of deep wells into karst aquifers are the most important conditioning factors (CFs). Arabameri et al.^13^ used three machine learning algorithms, i.e., an artificial neural network (ANN), bagging, and ANN-bagging. They reported that groundwater drawdown, ground use and ground cover, elevation, and lithology are the most important CFs.

Many studies have analyzed natural factors using mainly topographic or geological factors such as altitude, slope, slope aspect, curvature, fault, and ground use. Some studies have considered anthropogenic factors, including groundwater exploitation, ground use, roads, and subways. Among these factors, groundwater exploitation significantly affects ground sink. For example, Xu et al.^14^ investigated the ground sink CFs (GSCFs) of urban areas in Shanghai, China, and revealed that groundwater level decline, the construction of underground structures, building load, and dynamic load were related to ground sink. However, this study was limited as it considered only building foundations and rail tunnels as underground structures. Underground pipelines such as water pipes, sewer pipes, underground power pipes, natural gas pipelines, heat pipes, and communication lines did not receive sufficient attention.

As shown by these examples, topographic or geological factors have been used for susceptibility prediction and helped predict sinkholes or ground subsidence caused by natural factors. In Korea, the Samcheok and Yeongwol areas of Gangwon-do are underlain by limestone; thus, sinkholes may occur. Most of Seoul, an urban area, is underlain by granite and gneiss; thus, sinkholes are unlikely^15^. Instead, ground sink frequently occurs in urban areas. The Ministry of Environment reported that ground sinks in Korean cities were caused mainly by water and sewer pipes^16^. Specifically, 83.2% of the 3,328 ground sinks that occurred between 2010 and 2014 in Seoul were found to result from water and sewer pipes^8^. In light of this finding, this study aims to develop a machine learning model that determines the susceptibility to ground sink due to underground pipelines by analyzing the correlation between underground pipeline data and ground sink in urban areas.

This study proposes a method of identifying underground pipeline data correlated with ground sink and using machine learning to evaluate ground sink susceptibility. The proposed method has six steps: extraction of underground pipeline data attributes, data preprocessing, correlation analysis and significance evaluation, selection of GSCFs, susceptibility evaluation using machine learning, and validation. Finally, areas susceptible to ground sink are visualized by generating ground sink susceptibility maps (GSSMs).

The contributions of this study are as follows. First, datasets were constructed using points of ground sink occurrence, underground pipeline data, and drilling data. The points of ground sink occurrence were used as labels. Attributes that were estimated to be related to ground sink were extracted from the underground pipeline and drilling data. Datasets were constructed using both the labels and attributes. Second, this study focused on the impact of underground pipelines on ground sink. It was assumed that underground pipelines had a greater impact on ground sink in urban areas. Therefore, ground sink was evaluated using underground pipeline data.

This manuscript is organized as follows. “Study area” describes the natural and underground pipeline characteristics of the study area. “Data” explains the underground pipeline data, which were used as the raw data, and GSCFs. “Methods” describes the overall process in terms of correlation and significance, the machine learning model, accuracy evaluation, and validation. “Results” discusses the accuracy of the model and the GSSMs it was used to generate. Finally, “Conclusion” presents the conclusions.

## Study area

In this study, an urban area in South Korea where ground sink occurs frequently was selected to analyze the impact of ground sink generation by pipelines built under the urban area and evaluate the susceptibility. The urban area is a large city with a population exceeding one million and is considered to have a high risk of ground sink caused by underground pipelines because of its high concentration of underground pipelines. We selected five locations (A, B, C, D, and E) at which large ground sinks have occurred and then constructed underground pipeline data for areas 1 × 1 km in size, which were used for analysis. The data provision agreement makes it difficult to reveal the study area where the data were extracted. The key contribution of this study is the development of a machine learning model that uses underground pipeline data to classify ground sink susceptibility; the pipe type, pipe length, and pipe deterioration are more important factors in ground sink than the specific area. Therefore, the results of the study can be reported clearly even if the study area is not identified precisely.

## Data

### Raw data

**Figure 1:**
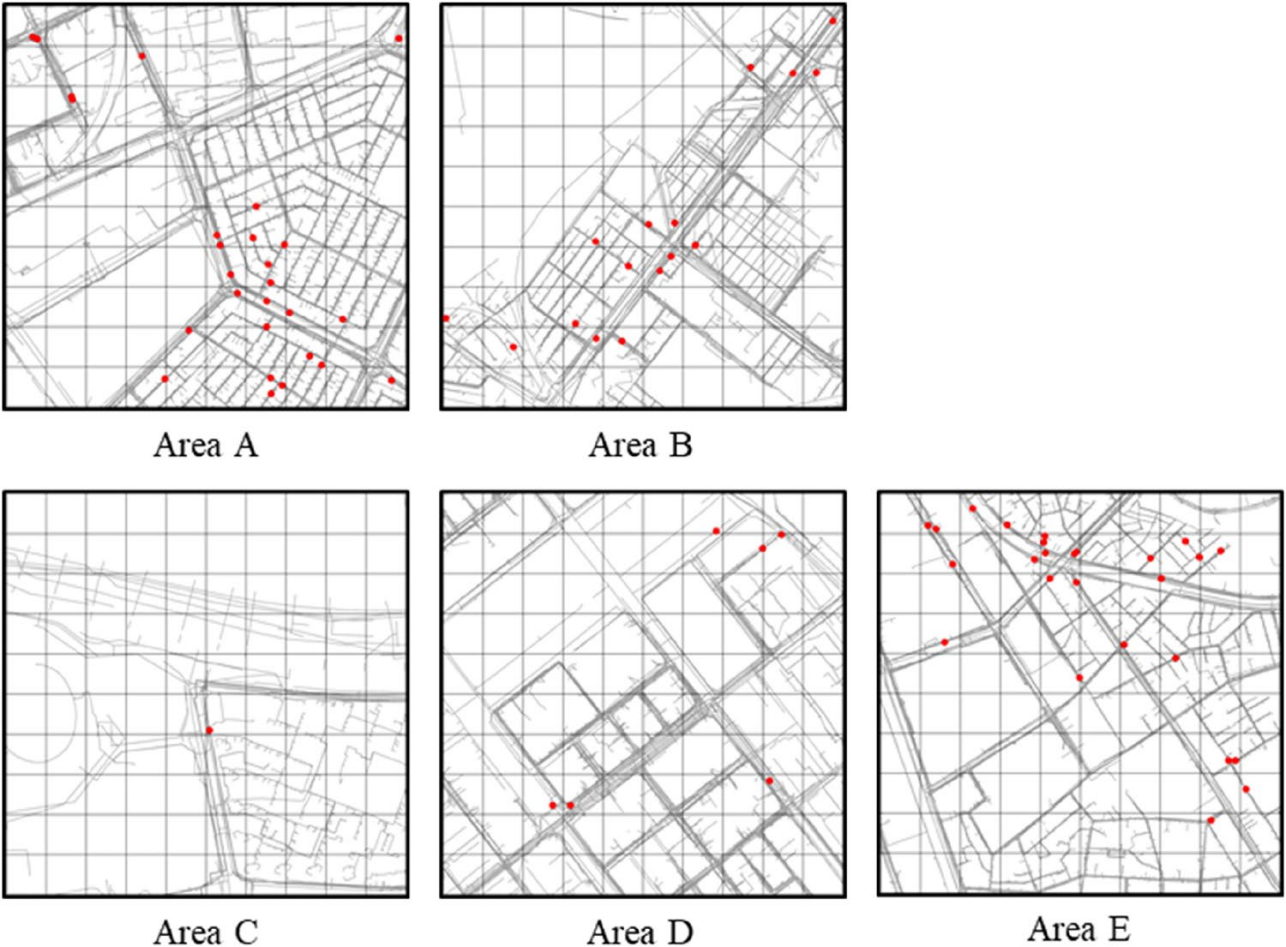
Points of ground sink occurrence.

Points of ground sink occurrence, underground pipeline data, and drilling data were used as the raw data. The points of ground sink are described using the *X* and *Y* coordinates, address, cause of ground sink, and recovery status of each location. The 77 points of ground sink occurrence in A, B, C, D, and E are shown in Fig. 1.

The underground pipeline data consist of data concerning water pipes, sewer pipes, underground power pipes, natural gas pipelines, heat pipes, and communication lines. The data contain geographic feature symbols, administrative region codes, management agency codes, and the installation date, diameter, material, and depth of each pipe.

The drilling data include borehole name, *X* and *Y* coordinates, groundwater depth, ground layer start and end depths, ground layer thickness, ground layer name, Unified Soil Classification System (USCS) class, and soil color. The drilling data contain information about the ground layer from the surface to the drilling end point. The soils were classified into 18 types according to the USCS.

### Selection of ground sink conditioning factors

**Figure 2:**
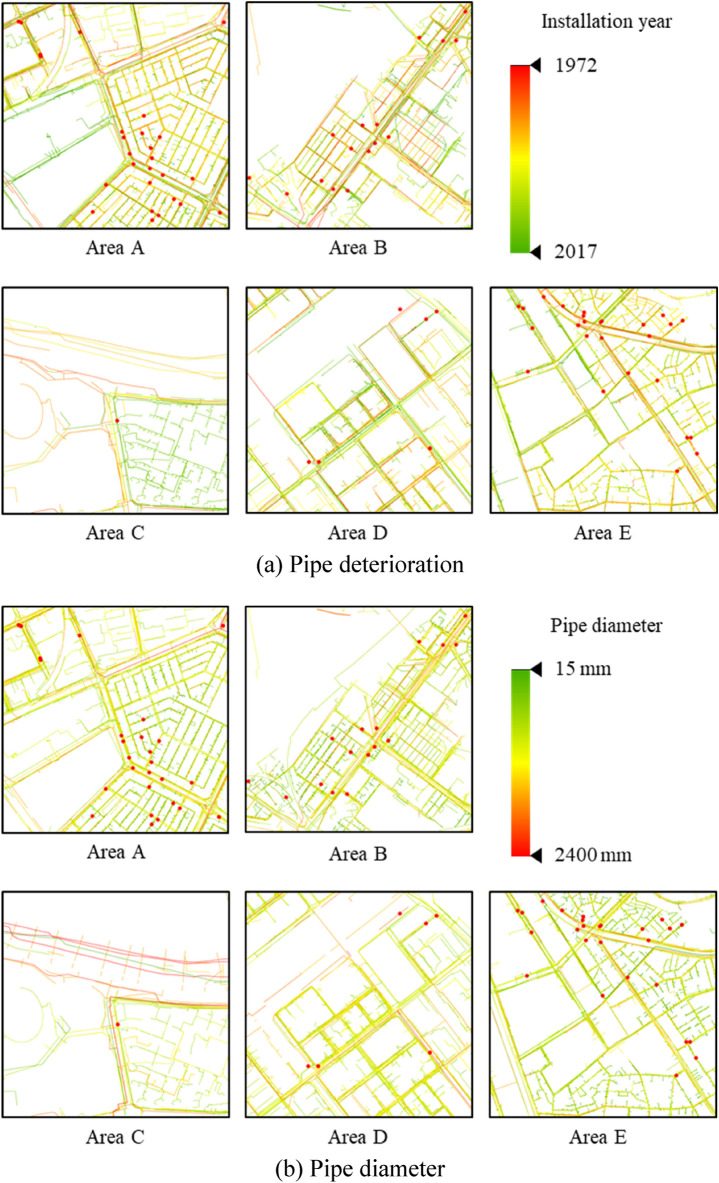

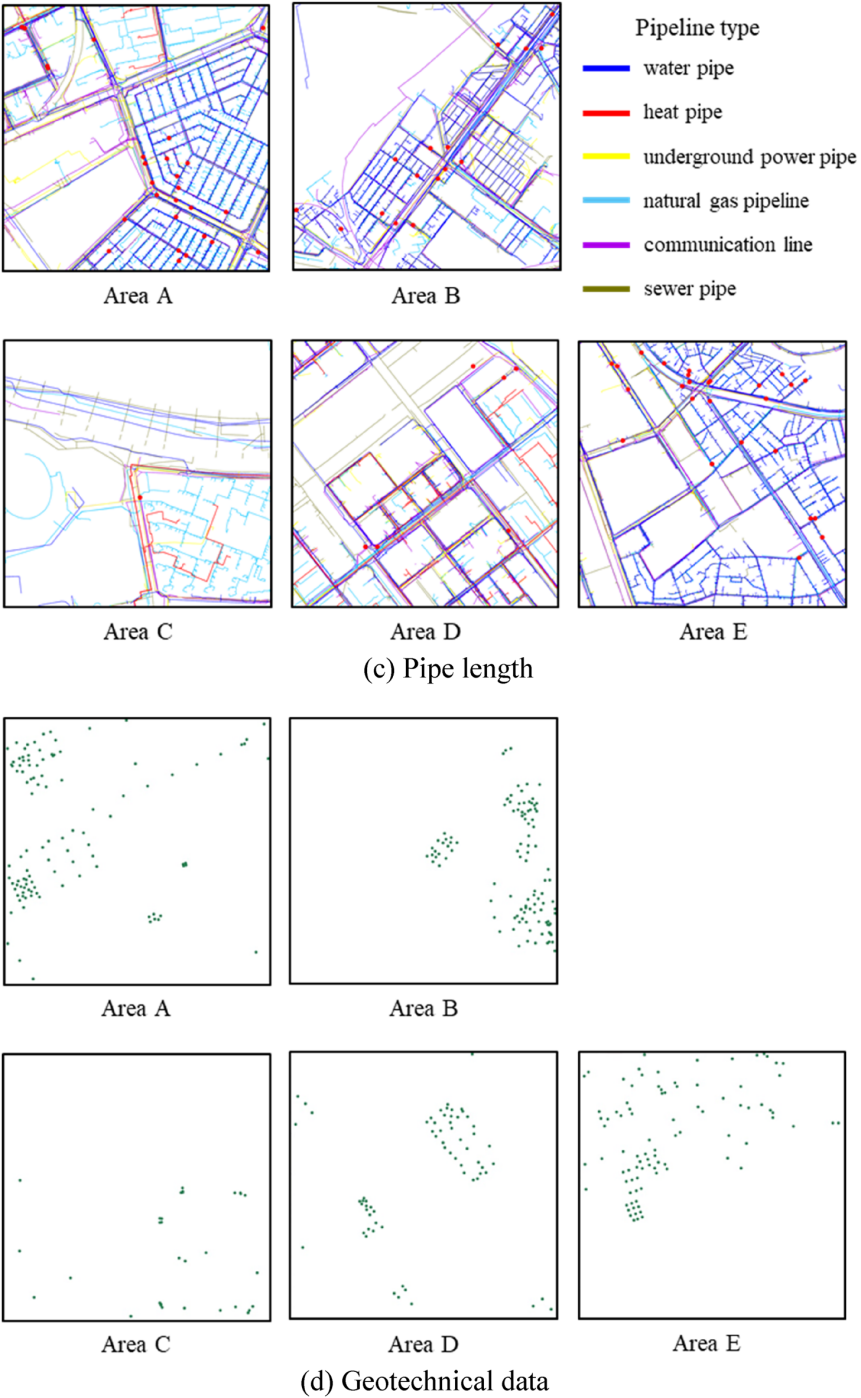
Ground sink conditioning factors.

Ground sink is related to groundwater level decline, underground utilities, and building load^14^. According to a report on urban areas, water and sewer pipe damage is among the major causes of ground sink^9,17^. This study used CFs related to pipelines as well as the attributes of underground pipeline data that have been used in previous studies. Geotechnical data were selected as the geological factor, and the pipe deterioration, pipe diameter, and pipe length were selected as the GSCFs. The data were initially analyzed assuming that pipe depth also affects ground sink. However, the analysis showed that pipe depth was not a meaningful characteristic because most pipelines were located with 3 m of the surface. Water pipes, sewer pipes, underground power pipes, natural gas pipelines, heat pipes, and communication lines associated with underground pipelines were considered as pipelines. The factors were selected according to the literature and expert knowledge. The selected factors are summarized as follows. First, the study area was divided into 100 $\times$ 100 m grids and split into 500 cells. Then the values of the selected factors were extracted or calculated for each cell. The results are shown in Fig. 2.

#### Pipe deterioration

The pipe installation year was among the attributes of underground pipelines used to indicate deterioration. Data for the period 1972 to 2017 were used, and pipes installed 30 years ago or more were determined to have deteriorated. According to “Service life of buildings etc.” (related to Subparagraph 1 of Paragraph 1 of Article 19) of the enforcement rules of the Local Public Enterprises Act, the service life of stainless steel, cast iron, and steel pipes is 30 years; that of galvanized steel pipes is 10 years, and those of other pipes are 20 to 30 years. Therefore, pipelines installed before 1992 were grouped into one category. In addition, the data for pipelines less than 30 years old were discretized to improve the machine learning prediction accuracy^18^. To prevent information loss during discretization, the learning results were compared after discretization was performed by dividing the data into 5- and 10-year intervals. Seven 5-year categories were obtained: 1972–1991, 1992–1996, 1997–2001, 2002–2006, 2007–2011, 2012–2016, and 2017. The four 10-year categories were 1972–1991, 1992–2001, 2002–2011, and 2012–2017. After the study area was divided into 500 cells in 100 × 100 m grids, the pipe length for each category was calculated for each cell and discretized into 100 m units. The results are shown in Fig. 2a.

#### Pipe diameter

The pipe diameter was selected from the attributes of the underground pipeline data. The pipe diameters were divided into four categories: 0–100 mm, 100–500 mm, 500–1000 mm, and 1000–2400 mm. After the study area was divided into 500 cells in 100 $\times$ 100 m grids, the pipe length for each pipe diameter category was calculated for each cell and discretized into 100 m units. The results are shown in Fig. 2b.

#### Pipe length

The pipe length was selected from the attributes of the underground pipeline data. Among the six types of underground pipelines, heat pipes were excluded because they had a negative correlation. Two methods were used to divide the category of pipeline length. First, the pipes were divided into five categories: water pipes, sewer pipes, underground power pipes, natural gas pipelines, and communication lines. Second, they were classified by material into group 1 (water pipes, natural gas pipelines), group 2 (underground power pipes, communication lines), and group 3 (sewer pipes). After the study area was divided into 500 cells in 100 $\times$ 100 m grids, the pipe length category for each cell was calculated. The pipe length was discretized into 100 m units. The results are shown in Fig. 2c.

#### Geotechnical data

**Figure 3:**
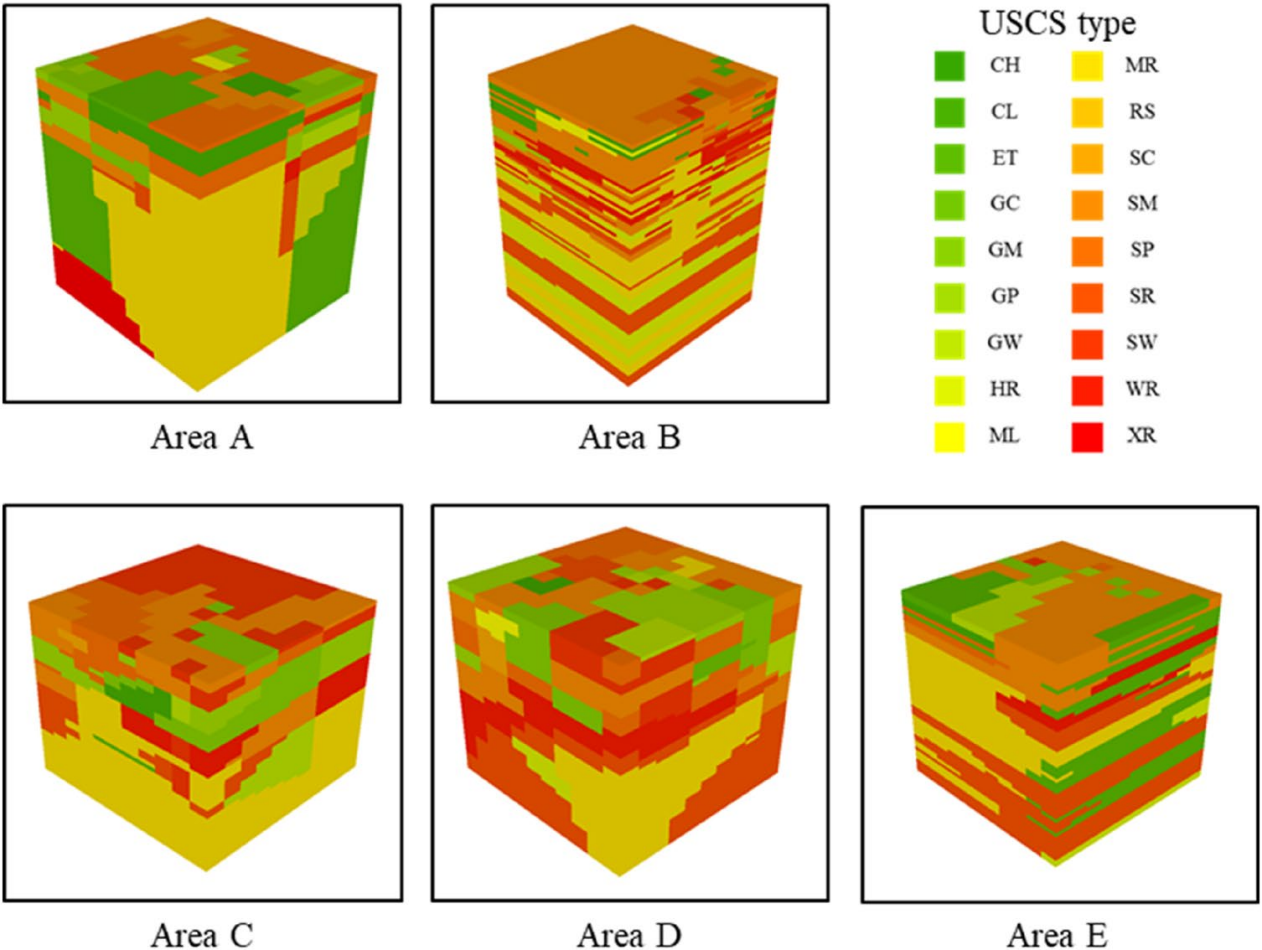
Drilling data visualized in ArcGIS.

The *X* and *Y* coordinates of each borehole, ground layer start and end depths, ground layer thickness, and USCS class, which are attributes related to drilling data, were used as geotechnical data. The drilling data were visualized in 3D using ArcGIS software (Fig. 3)^19^. The soils were classified into 18 types according to the USCS and arranged in the vertical direction (Table 1). Next, the geotechnical data of the study area were estimated using geostatistical interpolation by the Empirical Bayesian kriging 3D feature in ArcGIS. The estimated result was imported into a voxel layer. After the study area was divided into 500 cells in 100 $\times$ 100 m grids, the volume ratio of each soil type was calculated for each cell. The geotechnical data were analyzed down to the bedrock layer. The results showed that the bedrock layer is located at a depth of approximately 20 m. The volume ratio of each soil type was analyzed to depths of 20 m from the surface (Table 2). The geotechnical data were classified into five categories according to the permeability coefficient (Table 1). The results are shown in Fig. 2d.

**Table 1:** Geotechnical data.

Factor	Class
Geotechnical data	ET/GC/GM/GP/GW
	SC/SM/SP/SW
	CL/ML
	CH
	HR/MR/RS/SR/WR/XR

**Table 2:** Volume ratio of soil according to USCS.

Class	Area A (%)	Area B (%)	Area C (%)	Area D (%)	Area E (%)
CH	0	0	0	0	1
CL	7	15	1	0	4
ET	9	0	0	0	11
GC	0	0	0	1	0
GM	4	0	18	12	2
GP	19	0	12	22	1
GW	1	0	1	6	2
HR	2	16	0	0	0
ML	2	5	0	1	1
MR	13	23	18	0	14
RS	1	0	0	0	0
SC	0	1	1	3	3
SM	14	21	22	20	36
SP	15	2	9	17	4
SR	8	14	2	8	5
SW	0	1	7	5	2
WR	6	1	10	4	16
XR	0	0	0	0	0

## Methods

**Figure 4:**
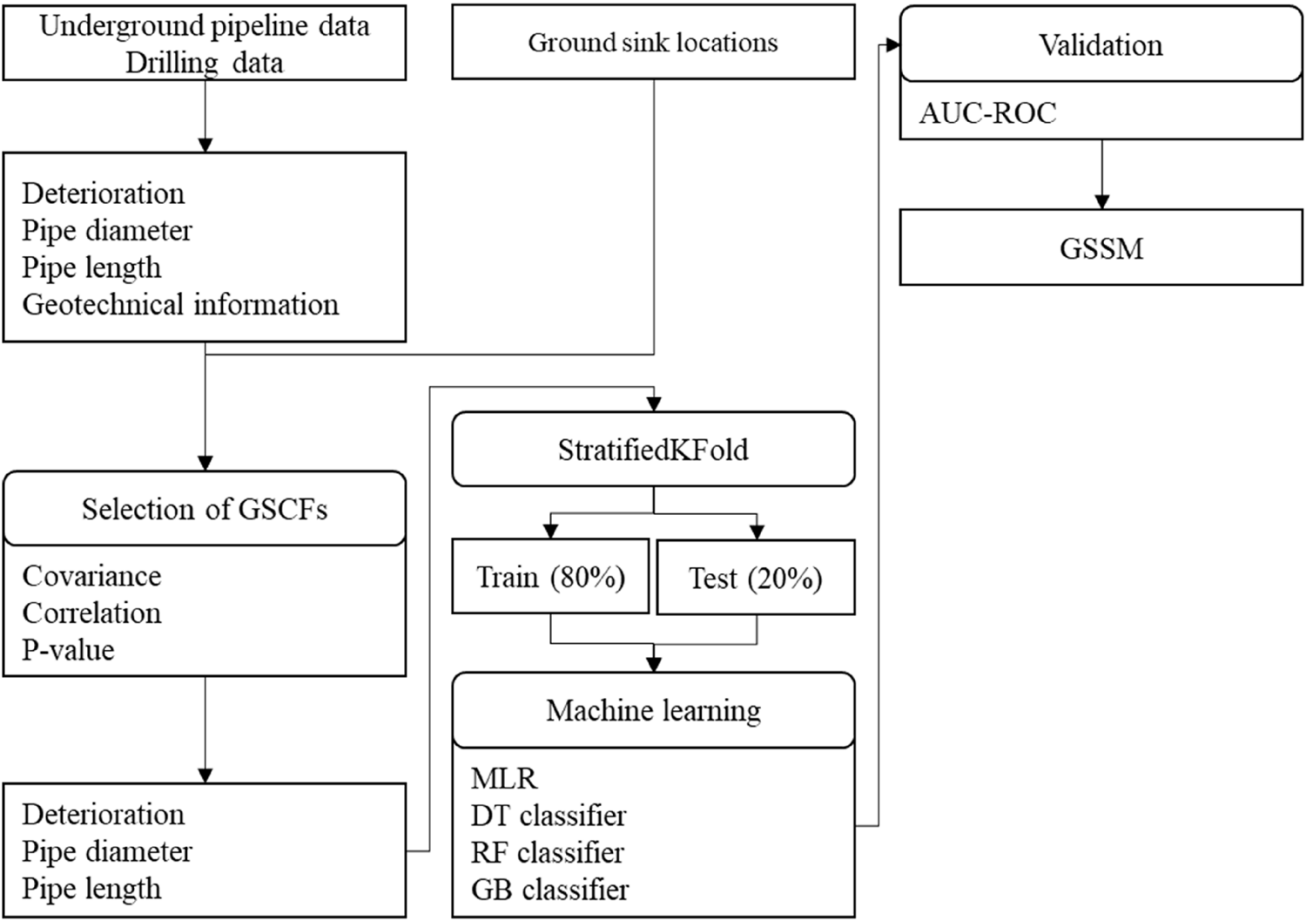
Flow chart of analysis.

This study conducted analyses in 1 $\times$ 1 km areas labeled A, B, C, D, and E. The 500 cells obtained by dividing these five areas into 100 $\times$ 100 m grids were analyzed. The flow chart in Fig. 4 illustrates the process. The underground pipeline and drilling data were used as raw data. From them, the deterioration, pipe diameter, pipe length, and geotechnical data for each type of pipe were extracted and used as input data. The training dataset was generated using the number of ground sinks and four GSCFs.

The sliding window method^20^ was used for correlation analysis and machine learning. Window sizes of 100 $\times$ 100 m, 200 $\times$ 200 m, and 300 $\times$ 300 m were used. The division into susceptibility classes was based on the number of ground sinks. When the window size was 100 $\times$ 100 m, the susceptibility classes were divided into three levels: 0, 1, and 2–5. For the 200 $\times$ 200 m window, they were divided into three levels: 0, 1, and 2–8 or four levels: 0, 1, 2–4, and 5–8. When the window was 300 $\times$ 300 m, they were divided into five levels: 0, 0.5, 1–1.5, 2–2.5, and 3–7.

Correlation analysis was performed after the datasets were divided. The correlation was calculated by standardizing the covariance between the number of ground sinks and GSCFs. Furthermore, the p-value was calculated after correlation analysis to determine the significance of the result.

The GSCFs were selected by correlation analysis and used as inputs to the machine learning model. When the data were input to the model, they were divided into training and test datasets at an 8:2 ratio using the fivefold cross-validation method, StratifiedKFold. After the data were divided, machine learning was performed using the training dataset. Multinomial logistic regression (MLR), decision tree (DT) classifier, random forest (RF) classifier, and gradient boosting (GB) classifier were used.

The susceptibility was evaluated by classifying the number of ground sinks, which is a machine learning prediction, by level. The susceptibility evaluation result was verified using the area under the receiver operating characteristic (AUC-ROC). In addition, GSSMs based on the susceptibility evaluation were generated.

### Correlation and significance

Correlations and significance can be identified by calculating the covariance, correlation, and p-value between the factors estimated by the GSCFs and the number of ground sinks.

Covariance is a linear function that indicates the correlation between two variables. Positive and negative covariance indicate positive and negative correlations, respectively. If the covariance is 0, *x* and *y* are independent variables with no correlation. The covariance is defined as the expected values of *x* and y multiplied by the deviation of *x* and *y*. It can be expressed as follows^21^:

1 $${\text{cov}}\left({\text{x}},{\text{y}}\right){=}\frac{1}{{\text{n}}{ - }{1}}\sum {(}{\text{x}}_{{\text{i}} \, }{- }\bar{\text{x}}\text{)(}{\text{y}}_{\text{i}} {- }\bar{\text{y}}{)}$$

The covariance is generally not considered to be useful for correlation analysis because the value varies with the magnitude of the measured values of *x* and *y*. Thus, the correlation coefficient ${r}_{xy}$ can be obtained by dividing the covariance by the standard deviations of *x* and *y*, ${s}_{x}$ and ${s}_{y}$. The correlation coefficient ranges from − 1 to 1 and is expressed as follows^21^:

2 $${\text{r}}_{\text{xy}} \, {=} \, \frac{\text{cov(x,y)}}{{\text{s}}_{\text{x}}{{\text{s}}}_{\text{y}}}$$

The p-value was used for significance testing. It represents the probability that an extreme sample mean is observed during sampling, assuming that the null hypothesis is true. If the p-value is below the threshold, the null hypothesis is rejected, and the alternative hypothesis is adopted^22^. The significance threshold was set to *P* ≤ 0.005^23^.

### Machine learning model

#### Multinomial logistic regression

Logistic regression is an algorithm that, despite its name, can be used for both regression and classification. When used for binary classification, logistic regression calculates the probability of belonging to each category as a value between 0 and 1 using a sigmoid function and predicts that an item belongs to the category if a specific probability is exceeded. In addition, logistic regression calculates the probability of belonging to each category when there are multiple categories. MLR is an extension of logistic regression in which there are three or more dependent variables^24^. It is implemented using the soft-max function. The level of ground sink is determined according to the number of ground sinks and used as a dependent variable. GSCFs are also used as independent variables. The probability of belonging to a category *y* is ${\text{P}}\left({\text{y}}\right){=}{\overrightarrow{\text{w}}}^{\text{T}}\overrightarrow{\text{x}}$. Here $\overrightarrow{\text{w}}$ is a weight, and $\overrightarrow{\text{x}}$ is an input feature value. This probability can be expressed using the log probability as ${\text{log}}\left({\text{P}}\left({\text{y}} \, {=} \, {\text{i}}\right)\right) {=} \, {\overrightarrow{\text{w}}}^{\text{T}}\overrightarrow{\text{x}} \, - \, {\text{log}}{\text{Z}}$. Because the sum of the calculated probabilities of belonging to each category must be 1, *Z* can be calculated as shown in Eqs. (3) and (4). The probability of belonging to each category is then expressed as Eq. (5)^25,26^.

3 $${1} \, {=}\sum_{{\text{i}}= \text{1} }^{\text{K}}{\text{P}}\left({\text{y}} {=}{\text{i}}\right){=}\frac{1}{{\text{Z}}}\sum_{{\text{i}}= \text{1} }^{\text{K}}{{\text{e}}}^{{\overrightarrow{\text{w}}}^{\text{T}}\overrightarrow{\text{x}}}$$

4 $$\text{Z} = \sum_{{\text{i}}= \text{1} }^{\text{K}}{{\text{e}}}^{{\overrightarrow{\text{w}}}^{\text{T}}\overrightarrow{\text{x}}}$$

5 $${\text{P}}\left({\text{y}}{=}{\text{i}}\right){=}\frac{{\text{e}}^{{\overrightarrow{\text{w}}}^{\text{T}}\overrightarrow{\text{x}}}}{{\sum }_{{\text{i}}= \text{1} }^{\text{K}}{{\text{e}}}^{{\overrightarrow{\text{w}}}^{\text{T}}\overrightarrow{\text{x}}}}$$

#### Decision tree classifier

DT classifier is a multistage decision-making method characterized by the identification of class membership using one or more decision functions continuously. It has a flow-chart-like tree structure that typically consists of a root node, a number of interior nodes, and a number of terminal nodes. The first and last nodes are called the root and nodes, respectively. Nodes that can no longer be divided into smaller groups are called terminal nodes^27,28^. A parent node generally branches into two child nodes such that the child nodes are purer than the parent node. The Gini index and entropy index are typically used to measure the impurity. Branching is performed by finding a branch condition under which the information gain, which is the difference in impurity between the parent and child nodes, is maximized. It proceeds in this way until the impurity is zero^29^. This algorithm is the most widely used because it is easier to implement and understand than other classification algorithms.

#### Random forest classifier

RF classifier is a method that compensates for the disadvantages of the DT classifier. Overfitting occurs when learning is performed until the impurity becomes zero in the DT classifier. To solve this problem, we use the RF classifier, an ensemble technique that combines tree classifiers. Each classifier is generated randomly. When the input data are given, the most frequently selected category is chosen after each classifier is applied^30^. RF classifier uses the Gini index as a measure of attribute selection. Here, the Gini index is used to measure the impurity of attributes for each category. If a random selection from training set *T* belongs to the category Ci, the Gini index is expressed as follows^31^:

6 $$\sum \sum_{{\text{j}} \ne \text{i}}{(}{\text{f}}{(}{\text{C}}_{\text{i}}, {\text{T}}{)/}\left|{\text{T}}\right|{)(}{\text{f}}{(}{\text{C}}_{\text{j}}, {\text{T}}{)/}\left|{\text{T}}\right|{)}$$

Here, $f({C}_{j}, T)/\left|T\right|$ is the probability that the selected case belongs to category C_i_.

#### Gradient boosting classifier

GB classifier also compensates for the disadvantages of the DT classifier. Like RF classifier, it uses ensemble techniques, but it differs in that classifiers are formed sequentially considering the error of the previous classifier. It uses GB, a process that improves classifiers by weighting them. First, an appropriate loss function is selected, and then DT classifier is used as a weak classifier. Finally, the weak classifiers are added sequentially in the direction of decreasing loss function. A classifier that has combined weak classifiers is called a strong classifier^26^. The algorithm can be derived as shown in Eqs. (7), (8), (9), (10), and (11) in Algorithm 1 for input training set ${{{(}{\text{y}}_{\text{i}}, {\text{x}}_{\text{i}}{)}}}_{1}^{\text{N}}$ and differentiable loss function ${\text{L}}{(}{\text{y}}, {\text{F}}{(}{\text{x}}{))}$, where the number of repetitions is *m* = 1, 2,$\cdots$, *M*^32^.

**Algorithm 1. Gradient boosting classifier algorithm.**

- Initialize model with a constant value:
  7 $${\text{F}}_{0}\left({\text{x}}\right){=}{{\text{arg}}{\text{min}}}_{\rho}{\sum }_{{\text{i}}= {1} }^{\text{N}}{\text{L}}{(}{\text{y}}_{\text{i}}, \rho {)}$$
- For *m* = 1 to *M*:
- Compute the so-called negative gradient:
  8 $${\widetilde{\text{y}}}_{\text{i}} \, {=}\text{ } - {\left[\frac{{\partial}{\text{L}}\left({\text{y}}_{\text{i}}{, }{\text{F}}\left({\text{x}}_{\text{i}}\right)\right)}{\partial {\text{F}}\left({\text{x}}_{\text{i}}\right)}\right]}_{{\text{F}}\left({\text{x}}\right) {=}{\text{F}}_{{\text{m}}-{1}}\left({\text{x}}\right)}{, } \, \text{for } \,\, {\text{i}} \, {=} \, {1, }{\text{N}}$$
- Fit $h(x;a)$ to the negative gradient:
  9 $${\text{a}}_{\text{m}} \, {=}{{\text{arg}}{\text{min}}}_{ \alpha {, } \beta }{\sum }_{{\text{i}}= {1} }^{\text{N}}{{[}{\widetilde{\text{y}}}_{\text{i}} {- } \beta \text{h}{(}{\text{x}}_{\text{i}}{; }{\text{a}}{)]}}^{2}$$
- Compute the multiplier ${\rho}_{\text{m}}$ by solving the following single-parameter optimization problem:
  10 $${\rho}_{\text{m}} {=}{{\text{arg}}{\text{min}}}_{\rho}{\sum }_{{\text{i}}= \text{1} }^{\text{N}}{\text{L}}{(}{\text{y}}_{\text{i}}{, }{\text{F}}_{{\text{m}}-{1}}\left({\text{x}}_{\text{i}}\right) \, {+} \, \rho \text{h}{(}{\text{x}}_{\text{i}}{;}{\text{a}}_{\text{m}}{))}$$
- Update the model:
  11 $${\text{F}}_{\text{m}}\left({\text{x}}\right) \, {=} \, {\text{F}}_{{\text{m}}-{1}}\left({\text{x}}\right) \, {+} \, {\rho}_{\text{m}}{{\text{h}}}_{\text{m}}{(}{\text{x}}{)}$$
- Output $${\text{F}}_{\text{M}}\left({\text{x}}\right)$$

### Accuracy evaluation and validation: AUC–ROC

**Figure 5:**
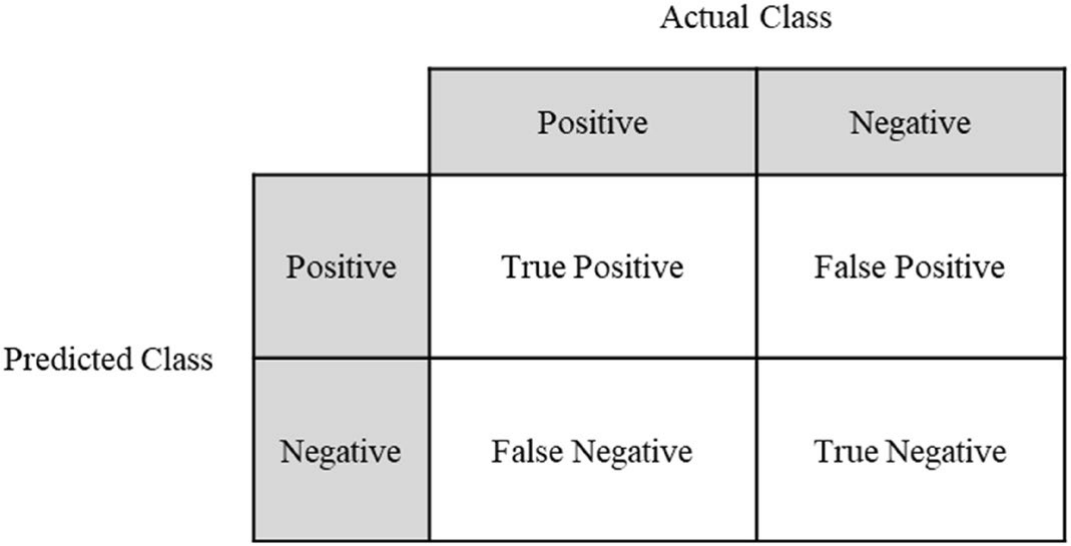
Confusion matrix.

The AUC–ROC was used to validate the results of the machine learning model. It is often used in machine learning^33^ and ground subsidence prediction^11,13,34^. The ROC curve uses the confusion matrix to check whether classification was performed correctly. Whether the actual classification result agrees with the inferred result is determined as shown in Fig. 5^33,35^.

The accuracy, sensitivity, and specificity can be calculated using Eqs. (12), (13), and (14), respectively, and the confusion matrix^33^.

12 $${\text{Accuracy}} \, {=}\frac{{\text{TP}} \, {+} \, {\text{TN}}}{{\text{TP}} \, {+} \, {\text{TN}} \, {+} \, {\text{FP}} \, {+} \, {\text{FN}}}$$

13 $${\text{Sensitivity}} \, {=} \, \frac{\text{TP}}{{\text{TP}} \, {+} \, {\text{FN}}}\text{(TPR:}\text{ true \, positive\, rate)}$$

14 $${\text{Specificity}} \, {=} \, \frac{\text{TN}}{{\text{TN}} \, {+} \, {\text{FP}}}\text{(TNR:}\text{ true \, negative\, rate)}$$

The ROC curve refers to the sensitivity graph for 1-specificity, which starts from the origin (0, 0) and ends at (1, 1). For model validation, the AUC of the ROC curve was used. An AUC-ROC value of 1 indicates perfect classification, and a value of 0.5 indicates that classification was not realized^35^.

## Results

### Correlation analysis

Correlation analysis was performed by calculating the covariance, correlation, and p-value between the number of ground sinks and GSCFs. It was applied to the deterioration, pipe diameter, pipe length, and geotechnical data using the sliding window method according to the window size and susceptibility classes. The correlation was considered to be distinct if the value was between 0.3 and 0.7, and a strong positive correlation was considered to exist if it was between 0.7 and 1. The result was considered significant for *p* < 0.005.

The deterioration obtained using 5- and 10-year units was compared. The results are shown in Table 3. The 10-year analysis generally showed a higher positive correlation. In the 5-year analysis, when the deterioration was low, the results tended not to be significant. The 10-year analysis results were all significant. The best result was obtained when the window size was 200 $\times$ 200 m.

**Table 3:** Deterioration correlation analysis: (a) 100 $\times$ 100 m, (b) 200 $\times$ 200 m, 3, (c) 200 $\times$ 200 m, 4, (d) 300 $\times$ 300 m.

Class	Covariance	Correlation	P-value
**(a) 100 × 100 m**			
1972–1991	0.57	0.3852	0.0000
1992–2001	0.28	0.2898	0.0000
2002–2011	0.20	0.2135	0.0000
2012–2017	0.10	0.2029	0.0000
1972–1991	0.57	0.3852	0.0000
1992–1996	0.11	0.1757	0.0001
1997–2001	0.13	0.2604	0.0000
2002–2006	0.17	0.2726	0.0000
2007–2011	0.01	0.0133	0.7661
2012–2016	0.09	0.1909	0.0000
2017	0.01	0.1240	0.0055
**(b) 200 × 200 m, 3**			
1972–1991	4.56	0.5352	0.0000
1992–2001	2.84	0.5100	0.0000
2002–2011	1.87	0.3594	0.0000
2012–2017	0.94	0.3353	0.0000
1972–1991	4.56	0.5352	0.0000
1992–1996	1.44	0.4059	0.0000
1997–2001	1.30	0.4690	0.0000
2002–2006	1.75	0.5043	0.0000
2007–2011	0.03	0.0088	0.8591
2012–2016	0.30	0.3442	0.0000
2017	− 0.01	− 0.0447	0.3694
**(c) 200 × 200 m, 4**			
1972–1991	5.25	0.5603	0.0000
1992–2001	3.09	0.5042	0.0000
2002–2011	2.12	0.3712	0.0000
2012–2017	1.02	0.3318	0.0000
1972–1991	5.25	0.5603	0.0000
1992–1996	1.52	0.3901	0.0000
1997–2001	1.46	0.4775	0.0000
2002–2006	1.98	0.5177	0.0000
2007–2011	0.06	0.0151	0.7619
2012–2016	1.01	0.3397	0.0000
2017	− 0.02	− 0.0469	0.3467
(**d) 300 × 300 mm**			
1972–1991	2.13	0.4943	0.0000
1992–2001	1.27	0.4567	0.0000
2002–2011	0.85	0.3162	0.0000
2012–2017	0.39	0.2634	0.0000
1972–1991	2.13	0.4943	0.0000
1992–1996	0.60	0.3310	0.0000
1997–2001	0.40	0.3540	0.0000
2002–2006	0.75	0.4216	0.0000
2007–2011	− 0.01	− 0.0050	0.9108
2012–2016	0.36	0.2579	0.0000
2017	0.00	0.0158	0.7251

The correlation analysis results for pipe diameter are shown in Table 4. The best results were obtained using the 200 × 200 m window. A positive correlation was observed in the pipe diameter range of 0–1000 mm; by contrast, no correlation appeared at 1000–2400 mm.

**Table 4:** Pipe diameter correlation analysis: (a) 100 $\times$ 100 m, (b) 200 $\times$ 200 m, 3, (c) 200 $\times$ 200 m, 4, (d) 300 $\times$ 300 m.

**Class**	**Covariance**	**Correlation**	**P-value**
**(a) 100 × 100 m**			
0–100	0.59	0.3670	0.0000
100–500	0.52	0.3078	0.0000
500–1000	0.19	0.3139	0.0000
1000–2400	− 0.01	− 0.0452	0.3129
**(b) 200 × 200 m, 3**			
0–100	5.36	0.5646	0.0000
100–500	4.16	0.4277	0.0000
500–1000	1.75	0.5175	0.0000
1000–2400	− 0.22	− 0.1287	0.0095
**(c) 200 × 200 m, 4**			
0–100	6.07	0.5818	0.0000
100–500	4.65	0.4347	0.0000
500–1000	1.99	0.5361	0.0000
1000–2400	− 0.25	− 0.1302	0.0087
(**d) 300 × 300 m**			
0–100	2.49	0.5310	0.0000
100–500	1.92	0.3916	0.0000
500–1000	0.74	0.4160	0.0000
1000–2400	− 0.08	− 0.0949	0.0339

The correlation analysis for pipe length was first performed for six types of underground pipelines: water pipes, sewer pipes, underground power pipes, natural gas pipelines, and communication lines. Heat pipes were excluded from further analysis because they were found to have a negative correlation, which was very small. The results for the other five types of underground pipelines of similar materials are summarized in Table 5. The positive correlations for pipes of similar materials was higher than that for different types of underground pipelines. The result for the 200 $\times$ 200 m windows was the best.

**Table 5:** Pipe length correlation analysis: (a) 100 $\times$ 100 m, (b) 200 $\times$ 200 m, 3, (c) 200 $\times$ 200 m, 4, (d) 300 $\times$ 300 m.

Class	Covariance	Correlation	P-value
**(a) 100 **$\times$** 100 m**			
W	52.09	0.3952	0.0000
U	21.51	0.2680	0.0000
N	25.79	0.2565	0.0000
C	41.66	0.3158	0.0000
S	28.21	0.3435	0.0000
Group 1	77.88	0.3762	0.0000
Group 2	63.17	0.3174	0.0000
Group 3	28.21	0.3435	0.0000
**(b) 200 **$\times$** 200 m, 3**			
W	4.16	0.5875	0.0000
U	1.12	0.3128	0.0000
N	2.14	0.4301	0.0000
C	2.60	0.4284	0.0000
S	2.08	0.5303	0.0000
Group 1	6.33	0.5764	0.0000
Group 2	3.76	0.4059	0.0000
Group 3	2.08	0.5303	0.0000
**(c) 200 **$\times$** 200 m, 4**			
W	699.47	0.5722	0.0000
U	212.42	0.3374	0.0000
N	376.75	0.4379	0.0000
C	451.07	0.4282	0.0000
S	349.77	0.5117	0.0000
Group 1	1076.22	0.5700	0.0000
Group 2	663.50	0.4150	0.0000
Group 3	349.77	0.5117	0.0000
**(d) 300 **$\times$** 300 m**			
W	182.29	0.5641	0.0000
U	21.61	0.2792	0.0000
N	99.48	0.4035	0.0000
C	115.05	0.3558	0.0000
S	92.46	0.4592	0.0000
Group 1	281.77	0.5551	0.0000
Group 2	169.99	0.3484	0.0000
Group 3	92.46	0.4592	0.0000

*W* water pipe, *N* natural gas pipeline, *U* underground power pipe, *C* communication line, *S* sewer pipe.

The geotechnical data of soils classified into 18 types according to the USCS were analyzed. The volume ratios were analyzed according to soil type from the surface to depths of 10, 15, and 20 m. The results are summarized in Table 6. There was no correlation whatsoever; the values were between − 0.3 and 0.3 in every category. Therefore, the geotechnical data were not classified as a GSCF.

**Table 6:** Geotechnical data correlation analysis: depths of (a) 10 m, (b) 15 m, (c) 20 m.

Class	Covariance	Correlation	P-value
**(a) 10 m**			
ET/GC/GM/GP/GW	0.00	0.0052	0.9162
SC/SM/SP/SW	− 0.06	− 0.0921	0.0640
CL/ML	0.09	0.1379	0.0054
CH	0.00	− 0.1115	0.0249
HR/MR/RS/SR/WR/XR	− 0.03	− 0.0784	0.1154
**(b) 15 m**			
ET/GC/GM/GP/GW	− 0.02	− 0.0218	0.6621
SC/SM/SP/SW	− 0.08	− 0.1349	0.0065
CL/ML	0.06	0.1362	0.0061
CH	0.00	− 0.1108	0.0257
HR/MR/RS/SR/WR/XR	0.04	0.0813	0.1025
**(c) 20 m**			
ET/GC/GM/GP/GW	0.02	0.0264	0.5958
SC/SM/SP/SW	− 0.10	− 0.1994	0.0001
CL/ML	0.04	0.1370	0.0057
CH	0.00	− 0.1076	0.0304
HR/MR/RS/SR/WR/XR	0.04	0.0644	0.1957

The correlation analysis indicated that the best results for pipe deterioration, pipe diameter, and pipe length were obtained when the window size was 200 $\times$ 200 m. A comparison of susceptibility levels of 3 and 4 revealed little effect on correlation. A comparison of the average correlation when the window size was 200 × 200 m showed that pipe length had the highest value of 0.5042, followed by pipe deterioration (0.4419) and pipe diameter (0.3556). In the USCS analysis, no positive correlation was found, with correlation values of − 0.3 to 0.3 in all categories.

### Model result

Machine learning was performed using MLR, DT classifier, RF classifier, and GB classifier for 16 cases, as shown in Table 7. Five-fold cross validation was performed using StratifiedKFold. The results are shown in Table 8.

**Table 7:** Number of categories of factors used in machine learning.

	Window size (m $\times$ m)	Susceptibility level	Deterioration	Pipe length	Pipe diameter
1	200 $\times$ 200	4	7	3	4
2	200 $\times$ 200	3	7	3	4
3	200 $\times$ 200	4	4	3	4
4	200 $\times$ 200	3	4	3	4
5	200 $\times$ 200	4	7	5	4
6	200 $\times$ 200	3	7	5	4
7	200 $\times$ 200	4	4	5	4
8	200 $\times$ 200	3	4	5	4
9	100 $\times$ 100	3	7	3	4
10	300 $\times$ 300	5	7	3	4
11	100 $\times$ 100	3	4	3	4
12	300 $\times$ 300	5	4	3	4
13	100 $\times$ 100	3	7	5	4
14	300 $\times$ 300	5	7	5	4
15	100 $\times$ 100	3	4	5	4
16	300 $\times$ 300	5	4	5	4

**Table 8:** Machine learning accuracy.

	MLR		DT classifier		RF classifier		GB classifier	
	Train	Test	Train	Test	Train	Test	Train	Test
1	0.7315	0.7062	0.7722	0.6815	0.7895	0.7037	0.8019	0.7087
2	0.7463	0.7309	0.7426	0.6790	0.7636	0.7333	0.7877	0.7407
3	0.7370	0.7210	0.7364	0.6765	0.7358	0.7136	0.7920	0.7012
4	0.7451	0.7309	0.7401	0.6963	0.7599	0.7383	0.7580	0.7383
5	0.7660	0.7235	0.7154	0.6716	0.7870	0.7111	0.8136	0.7160
6	0.7494	0.7235	0.7432	0.6914	0.8105	0.7383	0.7932	0.7432
7	0.7512	0.7309	0.7463	0.6864	0.7988	0.7210	0.7728	0.7284
8	0.7475	0.7333	0.7414	0.7037	0.7630	0.7407	0.7481	0.7358
9	0.8920	0.882	0.8910	0.888	0.8965	0.888	0.8890	0.886
10	0.6010	0.582	0.6405	0.574	0.6735	0.580	0.6520	0.568
11	0.8865	0.886	0.8910	0.884	0.8900	0.888	0.8950	0.890
12	0.6085	0.568	0.6060	0.546	0.6170	0.582	0.6435	0.571
13	0.8865	0.884	0.8890	0.878	0.8870	0.886	0.8870	0.886
14	0.6005	0.574	0.6510	0.568	0.6730	0.586	0.6120	0.570
15	0.8980	0.868	0.8875	0.885	0.9095	0.888	0.8930	0.888
16	0.6080	0.572	0.6395	0.572	0.6560	0.592	0.6650	0.578

Comparisons of the accuracy of each model showed that DT classifier had the lowest accuracy, whereas RT and GB classifier had the highest accuracy. The accuracy was lowest when the window size was 300 $\times$ 300 m and highest when it was 100 $\times$ 100 m. The the highest accuracy was obtained for dataset 11, where the window size was 100 $\times$ 100 m. However, at this window size, there was no actual classification accuracy because of overfitting to susceptibility classes with 0 accidents. Therefore, the results of datasets 1–8, with a window size of 200 $\times$ 200 m, were used.

The accuracy of the machine learning models for the training and test datasets was 0.7475 and 0.7333 for MLR, 0.7414 and 0.7037 for DT classifier, 0.7630 and 0.7407 for RF classifier, and 0.7932 and 0.7432 for GB classifier, respectively. The highest accuracy was obtained when GB classifier was used for dataset 6. Table 9 shows the hyperparameters used for training.

**Table 9:** Hyperparameters used in the machine learning model.

Model	Parameter
MLR	*C* = 0.001, multi_class = 'multinomial', solver = 'lbfgs', max_iter = 10,000
DT classifier	max_depth = 2
RF classifier	n_estimators = 500, max_depth = 3
GB classifier	n_estimators = 700, max_depth = 1, learning_rate = 0.01

### Model validation results

**Figure 6:**
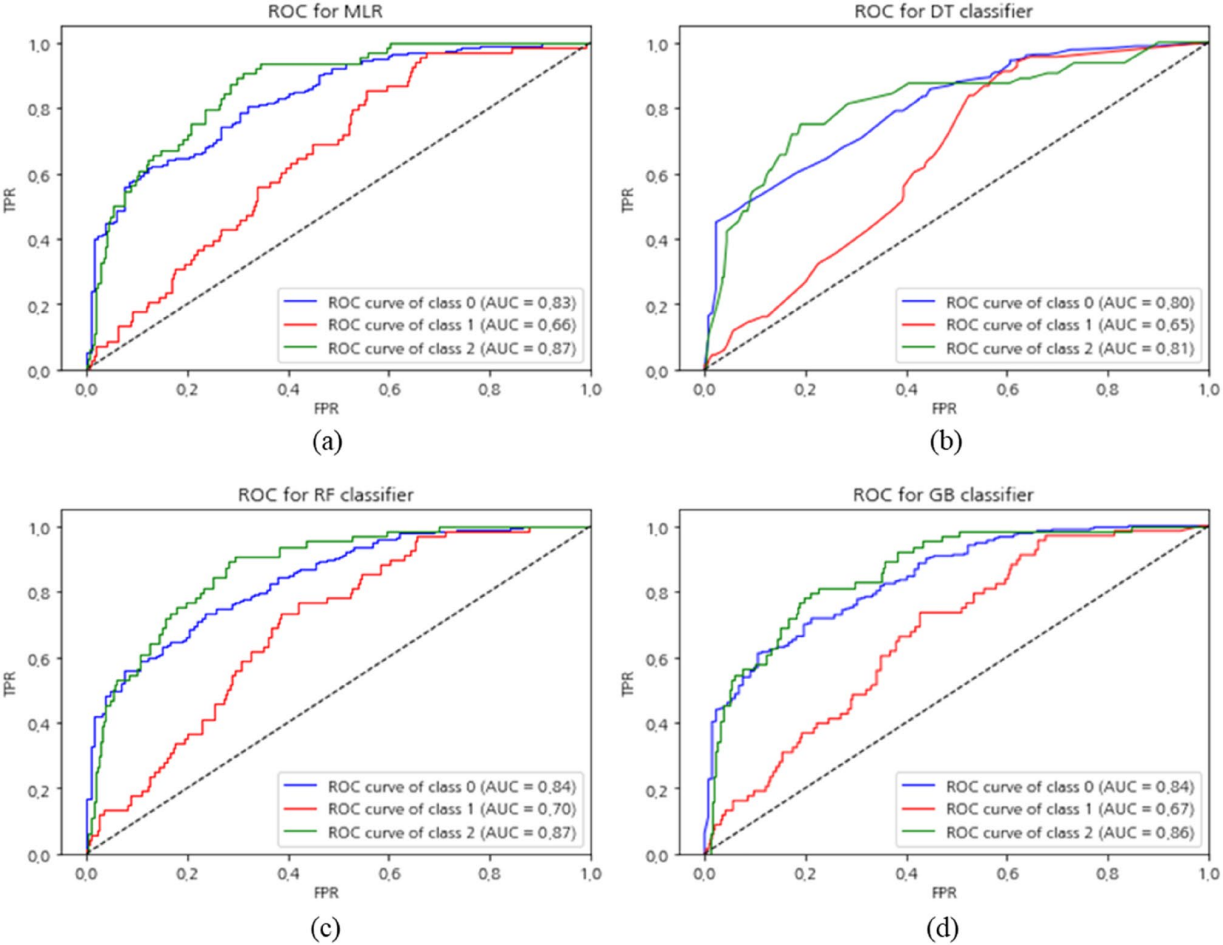
AUC–ROC: (**a**) MLR, (**b**) DT classifier, (**c**) RF classifier, (**d**) GB classifier.

The model was validated using AUC-ROC. The validation results for each machine learning model are shown in Fig. 6. After the ground sink susceptibility was identified as low, moderate, or high, the classification performance by category was evaluated. The AUC-ROC results were (0.83, 0.66, 0.87) for MLR, (0.80, 0.65, 0.81) for DT classifier, (0.84, 0.70, 0.87) for RF classifier, and (0.84, 0.67, 0.86) for GB classifier. Among the four models, RF and DT classifier had the highest and lowest AUC-ROC, respectively.

### Ground sink susceptibility maps

**Figure 7:**
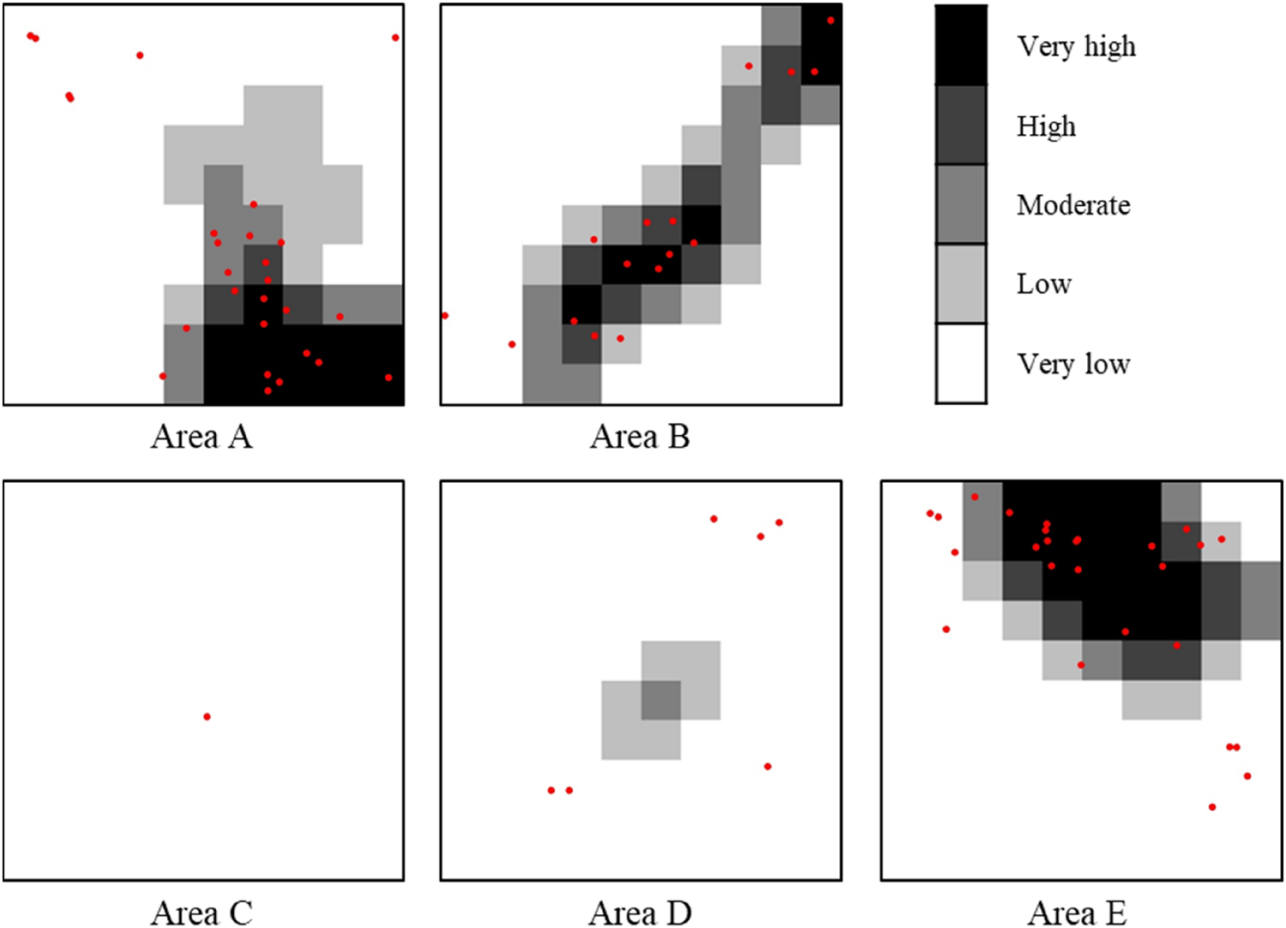
Ground sink susceptibility maps.

GSSMs were created using RF classifier. The machine learning model using GB classifier had a higher accuracy than that using RF classifier; however, the difference in accuracy was very small (0.0025). The GSSMs were created using RF classifier because of its greater reliability according to the AUC-ROC. The GSSMs are shown in Fig. 7. The ground sink susceptibility is expressed as very high, high, moderate, low, and very low. In the GSSMs, very low and very high risks are indicated by white and black, respectively. The most susceptible areas were A, B, and E, whereas C and D were generally safe. For A, B, and E, susceptibilities were predicted as close to very high in the lower right area in A, a diagonal area across B, and the upper center of E, where many ground sinks occurred. However, in areas C and D, where few ground sinks occurred, the susceptibilities were predicted to be very low or incorrectly predicted.

The GSSM creation process has several limitations and could be improved in several ways. The GSSMs have a low resolution because areas A, B, C, D, and E were divided into 100 cells each. The resolution could be improved if the areas were divided into a larger number of cells. However, if more cells were used, the cell size would decrease; thus, the number of ground sinks in each cell may be < 1. In this case, only two levels of susceptibility would be evaluated; thus, 100 $\times$ 100 m cells were used. To improve the resolution, it would be necessary to use data across a larger area or consider pixel-unit analysis by using a deep learning model based on a convolutional neural network (CNN).

## Conclusion

Ground sink in urban areas causes considerable environmental, social, and economic damage by destroying infrastructure. This study investigated the effects of the construction and operation of underground pipelines on ground sink in an urban area. For the study, the ground sink susceptibility in 1 $\times$ 1 km areas in an urban area in South Korea was evaluated using MLR, DT classifier, RF classifier, and GB classifier. The machine learning model was trained with GSCFs using fivefold cross validation by StratifiedKFold. Correlation analysis showed a low correlation between heat pipes and geotechnical data. Therefore, they were not included in the machine learning model because they did not seem to affect ground sink. A comparison of the average correlation values, the pipe length had the highest correlation (0.5042), followed by pipe deterioration (0.4419) and pipe diameter (0.3556). Among the four machine learning models, GB classifier showed the highest accuracy, whereas DT classifier showed the lowest accuracy. The difference in accuracy between GB classifier and RF classifier was not significant. However, when the models were verified using the AUC–ROC, RF classifier had the highest AUC, whereas DT classifier had the lowest AUC. A GSSM was created using RF classifier, which had high accuracy and reliability. Overall, the probability of ground sink was high in areas A, B, and E.

This study has the following limitations. The data used covered five 1 $\times$ 1 km areas; therefore, the data were often biased because of the small study area. Data changes during preprocessing are another limitation as it was found that the original data were not accurately represented as a result of data discretization and integer encoding. Because the study area was divided into 100 $\times$ 100 m grid units during machine learning, the locations of points at which ground sink occurred were not accurately reflected.

As demonstrated by Ministry of Environment data^8^ and previous studies^8,16^, ground sinks were generated mainly by underground pipelines. It has been found experimentally that in urban areas, the pipe type, pipe deterioration, and pipe length were significantly correlated with the frequency of ground sink. However, to improve the prediction accuracy of the model that determines the susceptibility to ground sink, it is necessary to consider not only human artifacts but also natural factors. Therefore, we plan to study an evaluation model that considers the effects of natural factors as well as underground pipes on ground sink. Furthermore, when evaluating the ground sink susceptibility, we will reduce the impact of preprocessing by applying a deep learning model based on a CNN, and thus use raw data without preprocessing.

## Data Availability

The data that support the findings of this study are available from the Korea Institute of Civil Engineering and Building Technology, but the availability of these data, which were used under license for the current study, is limited, and thus they are not publicly available. Data are, however, available from the corresponding author upon reasonable request and with permission from the Korea Institute of Civil Engineering and Building Technology.
